# Cohesin Loading Factor NIPBL Is Essential for MYCN Expression and MYCN-Driven Oncogenic Transcription in Neuroblastoma

**DOI:** 10.3390/cancers17162615

**Published:** 2025-08-09

**Authors:** Jee-Youn Kang, Kaitlyn A. Tremble, Philip Homan, Carol J. Thiele

**Affiliations:** 1 Pediatric Oncology Branch, Center for Cancer Research, National Cancer Institute, Bethesda, MD 20892, USA; jeeyoun.kang@nih.gov (J.Y.K.);; 2 CCR Collaborative Bioinformatics Resource, National Cancer Institute, Bethesda, MD 20892, USA; philip.homan@nih.gov; 3 Advanced Biomedical Computational Science, Frederick National Laboratory for Cancer Research, Frederick, MD 21702, USA

**Keywords:** MYCN, NIPBL, cohesion, neuroblastoma, transcription

## Abstract

High-risk neuroblastoma remains a major clinical challenge, with a five-year survival rate below 50% despite intensive multimodal therapies. MYCN amplification, a hallmark of high-risk disease, drives an aggressive transcriptional program that maintains undifferentiated and proliferative states in neuroblastoma cells. Given its central role in oncogenic transcription, MYCN represents an attractive therapeutic target; however, its undruggable nature has prompted efforts to identify upstream regulators or cofactors that sustain MYCN expression and oncogenic function. In this study, we investigate the role of the cohesin loading factor NIPBL in supporting the MYCN-driven transcriptional program. We demonstrate that elevated NIPBL expression is associated with undifferentiated, proliferative neuroblastoma cell states and poor clinical outcomes in neuroblastoma patients. Functionally, NIPBL depletion reduces MYCN mRNA and protein levels and induces widespread transcriptional reprogramming consistent with neuronal differentiation. These transcriptional changes are accompanied by decreased neuroblastoma cell proliferation and increased neuronal differentiation, reflecting impaired regulation of MYCN target genes upon NIPBL loss. Collectively, we have established a mechanistic link between NIPBL and the MYCN-driven transcriptome, highlighting NIPBL as a potential therapeutic vulnerability to promote differentiation in high-risk neuroblastoma.

## Simple Summary

Neuroblastoma is the most common extracranial solid tumors in children, and patients with high-risk disease have poor survival despite intensive multimodal therapies. MYCN amplification is a defining feature of high-risk neuroblastoma and drives a transcriptional program that maintains an undifferentiated and proliferative tumor state. Although MYCN is a well-established oncogenic driver, it remains a difficult therapeutic target. In this study, we investigated the role of NIPBL, a cohesin loading factor, in regulating MYCN-driven transcriptional programs. We found that elevated NIPBL expression is associated with poorly differentiated tumor phenotypes and worse clinical outcomes. Importantly, NIPBL depletion led to downregulation of MYCN expression and induced transcriptional reprogramming consistent with neuronal differentiation. These findings suggest that NIPBL sustains the oncogenic activity of MYCN and may serve as a tractable therapeutic vulnerability in high-risk neuroblastoma.

## 1. Introduction

Neuroblastoma is a pediatric tumor of the peripheral nervous system and accounts for 12–15% of childhood cancer-related deaths. Despite intensive multimodal therapies, outcomes for patients with high-risk neuroblastoma remain poor, with five-year overall survival rates below 50% [1]. Approximately 30% of high-risk neuroblastoma cases contain amplification of MYCN, a member of the MYC family of basic helix-loop-helix-leucine zipper (bHLH-LZ) transcription factors. MYCN plays a central role in driving an oncogenic transcriptional program [1]. Although MYCN is a compelling therapeutic target, direct inhibition is challenging due to its intrinsically disordered structure and lack of enzymatic activity. As a result, alternative strategies have focused on targeting cofactors critical to sustaining the MYCN oncogenic gene transcription program. [2,3,4,5,6].

The dynamic remodeling of DNA containing enhancer–promoter elements plays a critical role in regulating gene transcription, controlling cell lineage specification and differentiation [7,8]. Although initially recognized for its role in chromosome segregation during mitosis, the cohesin complex (composed of SMC1, SMC3, RAD21, and STAG1/2) is now known to regulate cell-type-specific enhancer–promoter interactions by extruding chromatin loops in an ATP-dependent manner [9]. NIPBL plays a critical role in guiding cohesin complex to open chromatin regions, positioning the cohesin at distal enhancers and gene promoters to initiate loop extrusion. This brings regulatory elements into proximity, enabling enhancer-bound transcription factors to activate gene expression by promoting RNA polymerase recruitment [10,11]. Mutations in the complex and its loading factor, NIPBL, have been identified in developmental disorders such as Cornelia de Lange syndrome (CdLS), as well as in pediatric cancers, including acute lymphoblastic leukemia (ALL) and Ewing sarcoma [12,13]. Instead of showing defects in chromosomal segregation, mutations in the cohesin have been found to impact transcriptional regulation. Recent studies have suggested that NIPBL interacts with transcription factors, such as the glucocorticoid receptor (GR) and Zfp609, to guide the cohesin to target loci, implicating transcription factor–NIPBL cooperation [14,15,16]. While transcription factor–NIPBL cooperation has been proposed as a mechanism for enhancer–promoter specificity in other systems, the role of NIPBL in neuroblastoma, particularly in MYCN-driven oncogenic transcription, remains largely unexplored.

In this study, we investigate the role of NIPBL in regulating the MYCN–driven transcriptional network in high-risk neuroblastoma. We demonstrate that elevated NIPBL expression is significantly associated with high-risk clinical features and poor patient outcomes. Moreover, we demonstrate that NIPBL is crucial for maintaining MYCN transcription and protein expression. Loss of NIPBL leads to widespread transcriptional reprogramming, characterized by the suppression of proliferative gene programs and the induction of neuronal differentiation markers. These findings identify NIPBL as a central regulator of the MYCN-driven oncogenic transcriptome, suggesting it as a potential therapeutic vulnerability in high-risk neuroblastoma.

## 2. Materials and Methods

### 2.1. Cell Lines and Reagents

The human neuroblastoma cell line SK-N-BE(2)-C (BE(2)-C), KCNR, and IMR-32 were obtained from the Pediatric Oncology Branch of the National Cancer Institute and have been genetically verified. Cells were grown in Roswell Park Memorial Institute (RPMI) 1640 medium with added fetal bovine serum (10% *v*/*v*), penicillin (100 IU/mL), streptomycin (100 mg/mL), and glutamine (2 mM). Cultures were tested biweekly for mycoplasma contamination using the MycoAlert kit (Lonza, Basel, Switzerland, #LT07–318), and all tests confirmed the absence of contamination.

### 2.2. Transient Transfection

Control siRNA (Dharmacon, Lafayette, CO, USA, #D-001206-13-20) and siRNAs targeting NIPBL (Dharmacon, #D-012980-19-0005 and # D-012980-21-0005) were transfected into neuroblastoma cells using Nucleofector electroporation (Lonza); Nucleofector^®^ electroporation kit V (Lonza, #VCA-1003) and program A-030 for BE(2)-C and KCNR. Nucleofector^®^ electroporation kit L (Lonza, #VCA-1005) and program C-005 for IMR32

### 2.3. Cell Proliferation and Viability Assay

To evaluate cell proliferation following NIPBL knockdown, BE(2)-C, KCNR, and IMR-32 cells transfected with siRNAs were seeded into 96-well plates. Cell growth was monitored over time using the IncuCyte ZOOM live-cell imaging system (Essen BioScience, Ann Arbor, MI, USA). Cell confluence, calculated using the integrated IncuCyte confluence algorithm, was used as a surrogate for cell number to track growth kinetics. At the experimental endpoint, cell viability was assessed using the CellTiter-Glo^®^ Luminescent Cell Viability Assay (Promega, Madison, WI, USA, #G9242), which measures ATP levels as an indicator of metabolically active cells. All conditions were tested in at least three technical replicates, and each experiment was independently repeated two to three times.

### 2.4. Protein Extraction and Western Blotting

Cells were collected from culture dishes using a rubber scraper and washed twice with ice-cold PBS. Cells were lysed in RIPA buffer (50 mM Tris-HCl pH 8, 150 mM NaCl, 1% Igepal-630, 0.5% Sodium Deoxycholate, 0.1% SDS, 1 mM EDTA) with HALT Protease and Phosphatase Inhibitor Single-Use Cocktail (Thermo Fisher Scientific, Waltham, MA, USA, #78442). Samples were centrifuged for 15 min at 20,000× *g* and 4°C, and the supernatant was analyzed for protein concentration using the Protein Assay Dye (Bio-Rad, #5000006). Protein lysates were brought to a concentration of 1 mg/mL in molecular water and Laemmli Sample Buffer (BioRad, Hercules, CA, USA, #1610747), denatured at 99°C for 10 min, and stored at −80°C before use.

Electrophoretic separation of proteins was carried out on 4–20% Mini-PROTEAN^®^ TGX™ Precast Protein Gels (Bio-Rad, #4561096) using a 25 mM Tris-HCl, 192 mM glycine, 1% SDS (pH 8.3) buffer. Following electrophoresis, proteins were transferred to a 0.45 mm nitrocellulose membrane (BioRad, #1620167) using the BioRad Trans-Blot^®^ Turbo Transfer System. Membranes were incubated first with blocking solution (5% nonfat milk or 5% BSA in TBST) for 1 hour at RT and then with primary antibodies (see Table 1) diluted in 5% BSA at 4°C overnight. Membranes were washed with Tris-Buffered Saline with Tween 20 (TBS-T), incubated with the appropriate HRP-conjugated secondary antibodies (see Table 1) for 1 hour at RT, and washed again with TBST. Membranes were visualized with SuperSignal™ West Femto Maximum Sensitivity Substrate (Thermo Scientific, #34096) on the BioRad ChemiDoc™ MP imaging system. Band intensities were quantified using Image Lab software (version 6.1).

### 2.5. RNA Sequencing

Total RNA was isolated from BE(2)-C cells 72 hours after transient transfected with siCont or two different siRNAs targeting NIPBL. Cells were collected and washed as above, and total RNA was isolated using RNeasy^®^ Plus Mini Kit (Qiagen, Germantown, MD, USA, #74134) according to the manufacturer’s instructions. Strand-specific whole transcriptome sequencing libraries were prepared using NEBNext Poly(A) mRNA Magnetic Isolation Module (New England Biolabs (NEB), Ipswich, MA, USA, #E7490), NEBNext Ultra II Directional RNA Library Prep Kit for Illumina (NEB #E7760) and NEBNext^®^ Multiplex Oligos for Illumina^®^ (96 Unique Dual Index Primer Pairs, NEB, E6440) as described in the manufacturer’s manual. Sequencing was performed on the Illumina NextSeq 2000 instrument using a 101 **×** 101 pair-end configuration.

Raw reads from RNA-seq data were processed using the CCR Collaborative Bioinformatics Resource (CCBR) RNA-seq pipeline [17]. In brief, reads were trimmed to remove adapter sequences and low-quality bases. Reads were aligned to the human genome(hg38) using STAR [18] in two-pass mode. Gene-level expression was quantified with RSEM [19] using the GENCODE hg38 version 30 annotation. Differentially expressed genes were identified using DESeq2 [19]. To investigate the biological pathways affected by NIPBL knockdown, Gene Set Enrichment Analysis (GSEA) was performed using the GSEA software (Broad Institute) with default parameters. The analysis was conducted using the normalized expression matrix and phenotype labels as input, and enrichment was assessed against the MSigDB gene sets [20]. Additionally, Gene Ontology (GO) enrichment analysis was conducted using the enrichGO function in the clusterProfiler R package 4.10.1, based on significantly differentially expressed genes. GO terms with adjusted *p*-values < 0.05 (Benjamini–Hochberg correction) were considered significant.

### 2.6. ChIP (Chromatin Immunoprecipitation) Sequencing

ChIP-seq was performed using the ChIP-IT High Sensitivity kit (Active Motif, #53040). Formaldehyde (1%, 15 min) fixed cells were sheared to 200–700 bp fragments using an Active Motif EpiShear Probe Sonicator. BE(2)-C cells were sonicated at 25% amplitude, pulse for 20 s on and 30 s off for a total sonication time of 16 min. Samples were immunoprecipitated overnight at 4°C with antibodies targeting MYCN (Active Motif, Cat # 61185) and NIPBL (Bethyl Laboratories Cat # A301-779). Active Motif ChIP-seq spike in chromatin (Active Motif Cat No. 53083) and Drosophila-specific histone variant H2Ac (Active Motif Cat No. 61686). Libraries prepared NEBNext Ultra II DNA Library Prep Kit (NEB, #E7645) and NEBNext^®^ Multiplex Oligos for Illumina^®^ (96 Unique Dual Index Primer Pairs, NEB, E6440) were multiplexed and sequenced on an Illumina NextSeq2000 machine.

Raw reads from ChIP-seq data were processed using the bioinfo-pf-curie/RNA-seq pipeline [21]. Briefly, reads were trimmed for adapter content and aligned to the hg38 genome using BWA-mem [22]. Spike-in sequences were aligned to dmelr6.32 and used to calculate a scaling factor. Low-quality reads, duplicate reads, and reads aligned to the spike-in reference were removed. Bigwig tracks were generated using spike-in normalized values. Peaks were identified using MACS2 [23]. High-confidence peaks were selected based on *p*-value (*p* < 1 × 10^−7^). Peaks were annotated using the HOMER software [24]. Sample Heatmap and profiles were generated using the deepTools2 suite [25]

### 2.7. Statistics

The statistical analysis used throughout this paper are specified in the appropriate results paragraphs and methods sections. For standard comparison, analysis were performed using a standard two-tailed Student’s *t*-test or one-way ANOVA on the software GraphPad Prism version 10.4.3.

## 3. Results

### 3.1. Aberrant Upregulation of NIPBL in Neuroblastoma Is Linked to Undifferentiated Cell States and Poor Clinical Outcomes

**Figure 1:**
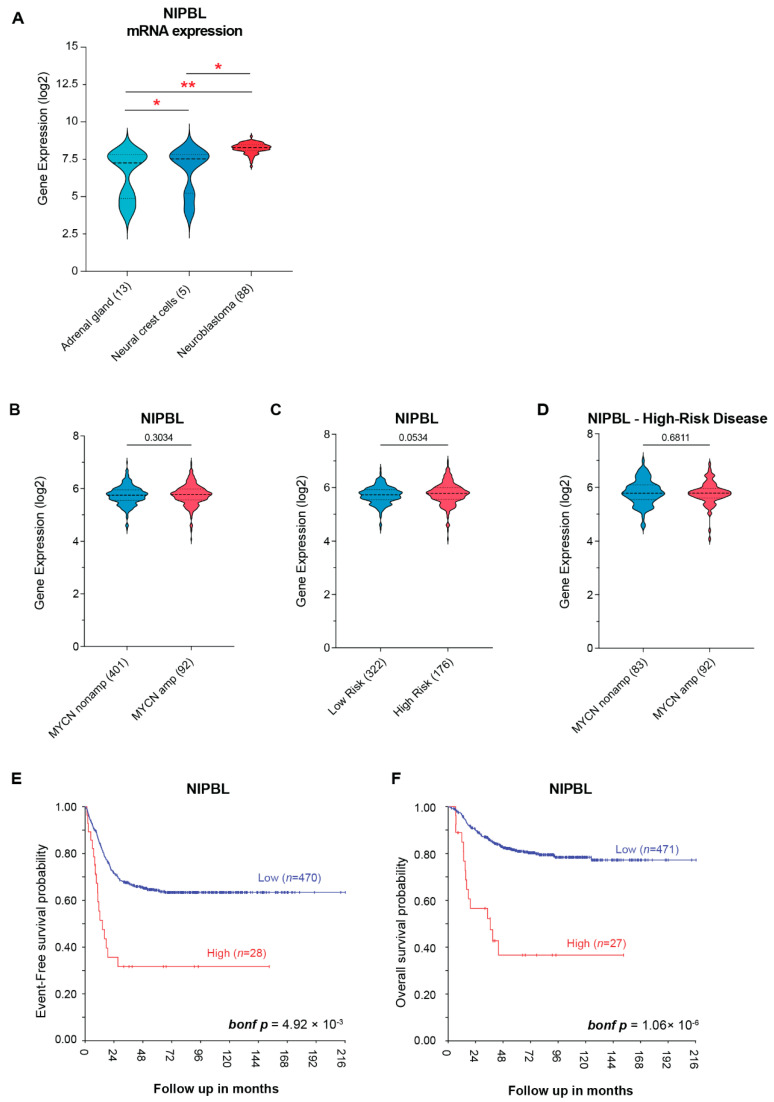
NIPBL expression is upregulated in neuroblastoma patients. (**A**) NIPBL mRNA expression levels across normal adrenal glands (*n* = 13), neural crest cells (*n* = 5), and primary neuroblastoma tumors (*n* = 88, Verteeg dataset), showing significantly increased expression in neuroblastoma patients; Welch’s two-tailed *t*-test. Asterisks indicate significance levels: *p* < 0.05 (*), *p* < 0.01 (**). (**B**) NIPBL mRNA expression in MYCN-amplified versus non-amplified neuroblastoma tumors (SEQC dataset); Welch’s two-tailed *t*-test. (**C**) NIPBL mRNA expression in low- and high-risk neuroblastoma tumors (SEQC dataset); Welch’s two-tailed *t*-test. (**D**) NIPBL mRNA expression in MYCN-amplified versus non-amplified tumors within the high-risk subgroup (SEQC dataset); Welch’s two-tailed *t*-test. (**E**) Kaplan–Meier analysis of event-free survival in neuroblastoma patients from the SEQC dataset (GSE49710), stratified into high (*n* = 28) and low (*n* = 470) NIPBL expression groups, demonstrates that high NIPBL expression is significantly associated with worse event-free survival (bonf *p*-value 4.92 × 10^−3^) (**F**) Kaplan–Meier analysis of overall survival in the same cohort, comparing high (*n* = 27) and low (*n* = 471) NIPBL expression groups, reveals a significant association between high NIPBL expression and poorer overall survival (bonf *p*-value 1.06 × 10^−6^). Kaplan–Meier survival analyses were performed using the R2: Genomics Analysis and Visualization Platform (http://r2.amc.nl accessed on 1 May 2025). High- and low-NIPBL expression groups were defined using the platform’s scan mode, which systematically evaluates all possible expression thresholds and selects the cutoff that yields the most statistically significant separation in survival outcomes. Survival differences were assessed using the log-rank test, and *p*-values were adjusted for multiple testing using the Bonferroni correction.

Neuroblastoma arises from the failure of neural crest cells to properly differentiate into the sympathoadrenal lineage, primarily due to dysregulated transcriptional programs [26]. Given that NIPBL is a critical regulator of enhancer–promoter looping, we sought to determine whether NIPBL expression is dysregulated during neuroblastoma tumorigenesis. We first compared NIPBL mRNA expression in neuroblastoma-related normal tissues, including neural crest cells and normal adrenal gland from the publicly available datasets with tumors from neuroblastoma patients using the R2: Genomics Analysis and Visualization Platform (https://r2.amc.nl accessed on 1 May 2025). NIPBL mRNA expression was significantly higher in the tumors of neuroblastoma patients compared to normal adrenal gland and neural crest cell samples, suggesting aberrant transcriptional activation during neuroblastoma tumorigenesis. Interestingly, NIPBL mRNA expression was higher in undifferentiated neural crest cells compared to that of normal adrenal gland tissues and was markedly upregulated in neuroblastoma tumors (Figure 1A).

To assess the clinical relevance of NIPBL in neuroblastoma, we analyzed the SEQC dataset (GSE49710) of primary neuroblastoma tumors with annotated clinical outcomes to determine whether NIPBL mRNA expression is associated with established clinical and molecular risk factors in neuroblastoma. NIPBL mRNA expression was not significantly different between MYCN-amplified and non-amplified neuroblastoma patients (Figure 1B). Again, no significant difference in NIPBL mRNA expression was observed between low-risk and high-risk patients based on the established risk classification (Figure 1C). Among high-risk patients, NIPBL expression did not significantly differ between MYCN-amplified and non-amplified tumors (Figure 1D).

However, Kaplan–Meier survival analysis revealed that high NIPBL mRNA expression is significantly associated with worse event-free survival (bonf *p*-value = 4.92 × 10^−3^; Figure 1E) and overall survival (bonf *p*-value = 1.06 × 10^−6^; Figure 1F). These results suggest that elevated NIPBL mRNA expression is associated with poor clinical outcomes of neuroblastoma patients.

### 3.2. NIPBL Is Essential for Neuroblastoma Proliferation

**Figure 2:**
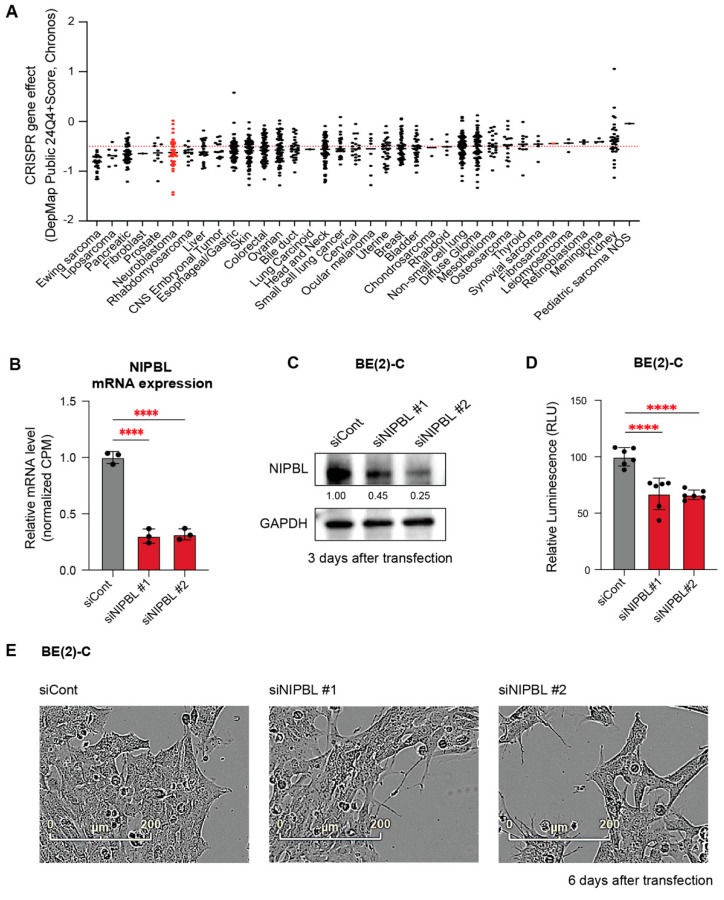
NIPBL is essential for neuroblastoma cell proliferation. (**A**) DepMap Chronos dependency scores across different tumor types were analyzed. A dependency score below −0.5 indicates that the target gene is essential for the survival of the cell line. Neuroblastoma cell lines are among the tumor types that show dependency on NIPBL. (**B**) Downregulation of NIPBL mRNA expression was confirmed with RNA-seq results from NIPBL-depleted BE(2)-C cells after 3 days of transfection. Expression levels are shown relative to siCont. Statistical significance was determined using one-way ANOVA. “****” indicates statistical significance with *p* < 0.0001. (**C**) Western blot confirming NIPBL protein depletion in BE(2)-C cells with two siRNAs. GAPDH served as a loading control. (**D**) CellTiter-Glo assay demonstrating significantly reduced viability of BE(2)-C cells six days after NIPBL depletion. (**E**) Phase-contrast images showing decreased cell confluency in BE(2)-C cells six days following NIPBL depletion.

We sought to determine whether NIPBL plays a direct functional role in the survival and proliferation of neuroblastoma cells. To assess functional dependency, we analyzed genome-wide CRISPR-Cas9 knockout screens from the Cancer Dependency Map (DepMap https://depmap.org accessed on 1 May 2025), which quantifies gene essentiality using a Chronos dependency score (dependency score ≤ –0.5 indicates that the targeted gene is essential for cell viability). Neuroblastoma was identified as one of the top ten tumor types most dependent on NIPBL (Figure 2A).

To experimentally validate this dependency, we used two independent small interfering RNAs (siRNAs) to deplete NIPBL in MYCN-amplified neuroblastoma cells, BE2C, KCNR, and IMR-32. Knockdown efficiency was confirmed by RNA-seq and Western blot analysis at the indicated time points in BE(2)-C cells (Figure 2B,C). Consistent with the dependency identified from DepMap, cell viability and cell confluency were significantly reduced in BE(2)-C cells six days after NIPBL depletion, as measured by CellTiter-Glo and IncuCyte ZOOM live-cell imaging (Figure 2D,E). Similar decreases in cell viability were observed in KCNR and IMR-32 cells (Figure S2A–F, in the supplement materials), indicating that the cohesin loading factor NIPBL is essential for the proliferation of MYCN-amplified neuroblastoma cells (Figure S1, in the supplement materials).

### 3.3. NIPBL Is Essential for Maintaining Oncogenic Transcriptional Programs in Neuroblastoma Cells

**Figure 3:**
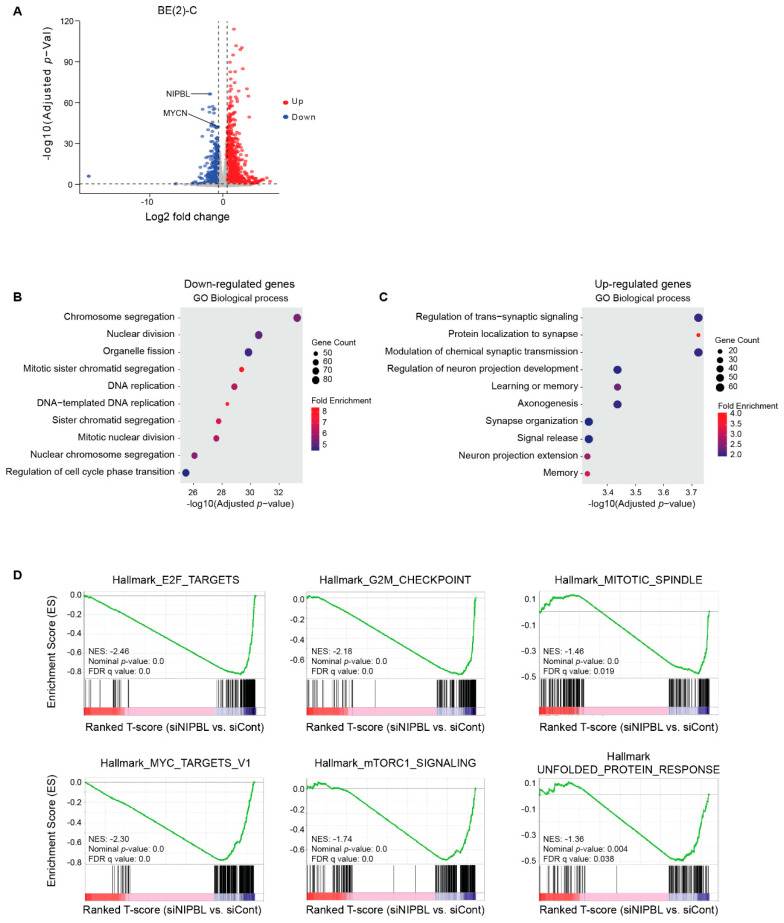
NIPBL maintains neuroblastoma oncogenic transcriptome. (**A**) Volcano plot showing differentially expressed genes in BE(2)-C cells transfected with siRNAs targeting NIPBL compared to control siRNA (log2 fold change > ±1.5, *p*-value < 0.05). Up- and down-regulated genes are represented by red and blue symbols. Genes with no significant expression change are represented by gray symbols (**B**) Gene Ontology (GO) enrichment analysis of downregulated genes upon NIPBL knockdown reveals strong enrichment in cell cycle–related biological processes, including DNA replication, mitosis, and chromosome segregation. (**C**) GO enrichment analysis of upregulated genes shows significant enrichment in neuronal differentiation–related processes such as synapse organization, axonogenesis, and trans-synaptic signaling. (**D**) Gene Set Enrichment Analysis (GSEA) plots show significant negative enrichment of hallmark gene sets associated with proliferation and oncogenic activity, including E2F targets, the G2M checkpoint, MYC targets, the mitotic spindle, the unfolded protein response, and mTORC1 signaling, following NIPBL depletion.

To assess the global transcriptional effects of NIPBL knockdown, we performed RNA-seq analysis on BE2C neuroblastoma cells three days of NIPBL depletion. Differential gene expression analysis revealed 2213 genes with significant changes (fold change >1.5 or <−1.5; *p* < 0.05), including 790 downregulated and 1423 upregulated genes (Figure 3A). Gene Ontology (GO) analysis of downregulated genes revealed strong enrichment in pathways associated with cell cycle progression, including DNA replication, chromosomal segregation, and mitotic cell division, consistent with a loss of proliferative capacity and induction of cell cycle arrest (Figure 3B). In contrast, upregulated genes were enriched in neuronal processes, including synaptic signaling, axonogenesis, and regulation of trans-synaptic signaling, reflecting a shift toward neuronal differentiation (Figure 3C). Gene Set Enrichment Analysis (GSEA) further demonstrated significant downregulation of hallmark pathways associated with proliferation, including E2F targets, G2/M checkpoint, mitotic spindle assembly, and MYC target gene sets (Figure 3D). Additionally, pathways supporting oncogenic metabolism, such as the unfolded protein response and mTORC1 signaling, were suppressed following NIPBL depletion (Figure 3D). Together, these results suggest that NIPBL maintains the MYCN-driven transcriptional program that promotes cell proliferation and a stem-like identity in neuroblastoma cells.

### 3.4. NIPBL Co-Occupies MYCN-Driven Enhancers in MYCN-Amplified Neuroblastoma

**Figure 4:**
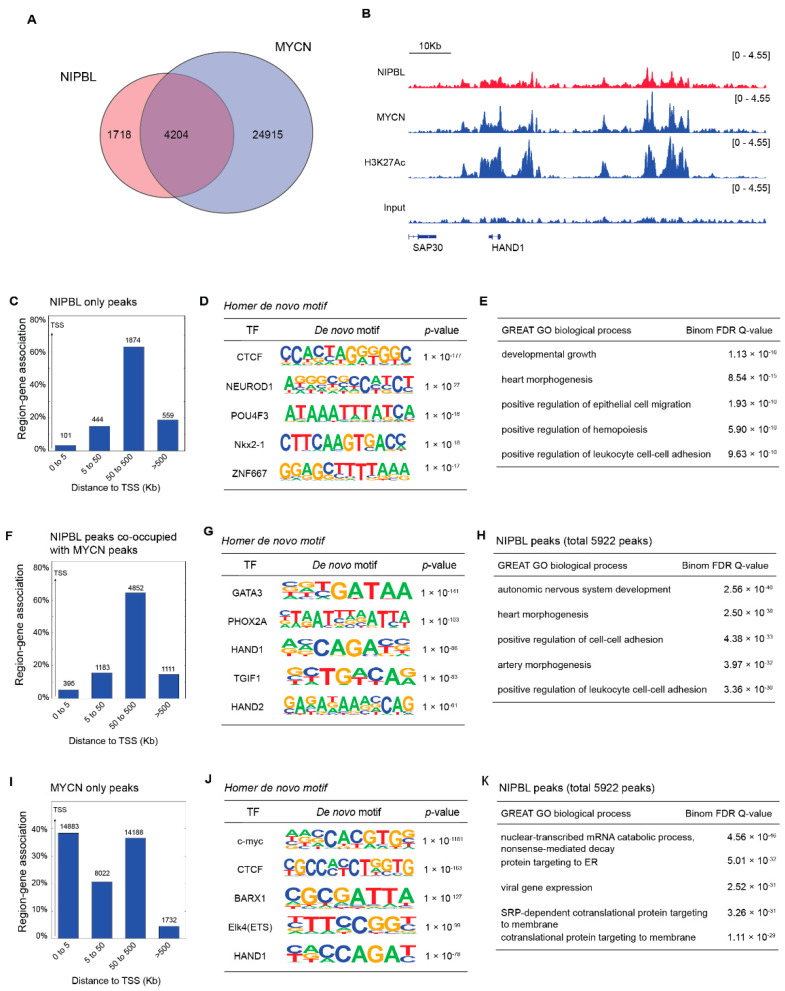
NIPBL co-occupies MYCN-bound enhancers in the neuroblastoma genome. (**A**) Venn diagram showing the overlap between NIPBL and MYCN ChIP-seq peaks in BE(2)-C cells. The overlapping peaks were identified using the Intervene tool. (**B**) Representative Integrative Genomics Viewer (IGV) browser tracks of the HAND1 locus showing co-occupancy of NIPBL and MYCN at promoter and enhancer regions in BE(2)-C cells. Enhancer and promoter elements were defined based on H3K27ac ChIP-seq signal intensity. (**C**) GREAT peak distribution analysis of NIPBL only peaks relative to transcription start sites (TSS) (**D**) HOMER de novo motif analysis of NIPBL-only peaks. (**E**) GREAT Gene ontology (GO) analysis showing NIPBL-only peaks. (**F**) GREAT peak distribution analysis of NIPBL/MYCN co-occupied peaks relative to transcription start sites (TSS). (**G**) HOMER de novo motif analysis of NIPBL/MYCN co-occupied peaks. (**H**) GREAT Gene ontology (GO) analysis showing NIPBL/MYCN co-occupied peaks. (**I**) GREAT peak distribution analysis of MYCN-only peaks relative to transcription start sites (TSS) (**J**) HOMER de novo motif analysis of MYCN-only peaks. (**K**) GREAT Gene ontology (GO) analysis showing MYCN-only peaks.3.5 NIPBL sustains MYCN expression to suppress neuronal differentiation programs in MYCN amplified neuroblastoma.

Although NIPBL is known to associate with the enhancers and promoters of cell-type-specific genes, it does not recognize specific DNA sequences on its own [27]. Instead, NIPBL colocalizes with lineage-defining transcription factors at regulatory elements [14,15,16,28]. These studies suggest that certain transcription factors can recruit NIPBL to facilitate cohesin loading at target enhancers and promoters. Given that MYCN is a key oncogenic driver in high-risk neuroblastoma and governs a broad transcriptional network, we hypothesized that NIPBL may colocalize with MYCN in the neuroblastoma genome. To test this, we performed genome-wide ChIP-seq for NIPBL and MYCN in the MYCN-amplified neuroblastoma cell line BE(2)-C. We identified 5922 NIPBL peaks, of which 4204 overlapped with MYCN peaks (Figure 4A), suggesting widespread genomic co-occupancy of NIPBL with MYCN. Representative ChIP-seq signal tracks show colocalization of NIPBL and MYCN at both the promoter and enhancers of the HAND1 gene locus (Figure 4B). GREAT analysis of peak distribution revealed that the majority of NIPBL peaks—regardless of MYCN colocalization—were enriched at distal regulatory elements, beyond ± 5 kb from transcriptional start sites (Figure 4C,F), consistent with NIPBL’s role at enhancers. De novo motif analysis using HOMER revealed distinct transcription factor motif enrichment patterns between the two categories of NIPBL peaks. NIPBL peaks not bound by MYCN were enriched for motifs of transcription factors involved in neuronal differentiation, such as NEUROD1 and POU4F3. In contrast, NIPBL peaks that overlapped with MYCN binding were significantly enriched for motifs recognized by core regulatory circuitry (CRC) transcription factors critical to neuroblastoma cell identity, including GATA3, PHOX2A HAND1, and HAND2 (Figure 4D,F) [29]. In contrast, MYCN peaks that did not colocalize with NIPBL were enriched at both promoters and distal regulatory regions and contained MYC binding motifs as well as motifs for transcription factors associated with broader developmental programs, including BARX1 and ELK4 (Figure 4J).

GREAT GO analysis revealed that genes associated with NIPBL peaks were enriched in biological processes, including lineage-specific developmental processes such as heart morphogenesis and the autonomic nervous system (Figure 4E,H). Conversely, genes linked to MYCN-only peaks were enriched for functions related to mRNA metabolism, protein targeting, and viral gene expression (Figure 4K).

Together, these results suggest that NIPBL is preferentially recruited to MYCN-bound enhancer regions enriched for CRC transcription factor motifs. In addition, NIPBL is also found at enhancers that have binding motifs for transcription factors involved in neuronal differentiation.

### 3.5. NIPBL Sustains MYCN Expression to Suppress Neuronal Differentiation Programs in MYCN Amplified Neuroblastoma

**Figure 5:**
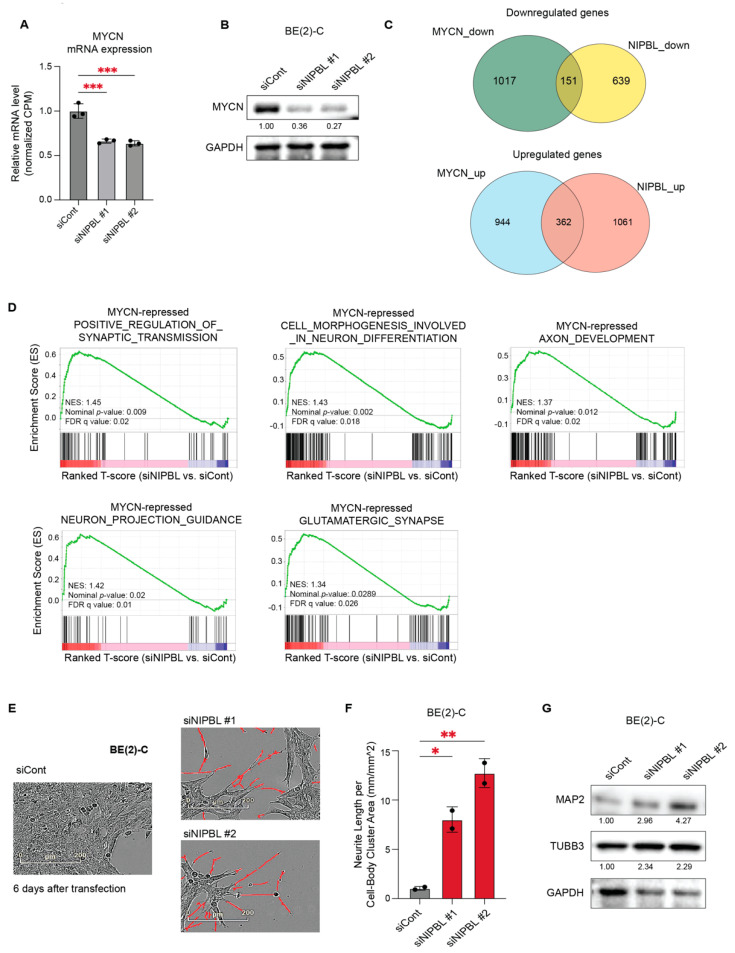
NIPBL regulates MYCN expression and represses neuronal differentiation programs in MYCN-amplified neuroblastoma cells. (**A**) Downregulation of MYCN mRNA expression was identified by RNA-seq analysis of NIPBL-depleted BE(2)-C cells. Expression levels are shown relative to the siCont. Statistical significance was determined using one-way ANOVA. *p* < 0.001 (***). (**B**) Western blot analysis showing reduced MYCN protein levels upon NIPBL knockdown. GAPDH serves as a loading control. (**C**) Venn diagrams showing the overlap of differentially expressed genes between MYCN and NIPBL knockdown RNA-seq datasets. (**D**) GSEA enrichment plots for gene sets previously identified as MYCN-repressed neuronal differentiation pathways. NIPBL knockdown significantly derepresses gene sets involved in positive regulation of synaptic transmission, neuron projection guidance, cell morphogenesis in neuron differentiation, axon development, and glutamatergic synapse. Each gene set shows a positive enrichment score (NES) with significant nominal *p*-values and FDR q-values. (**E**) Phase-contrast image showing enhanced neurite-like extensions in BE(2)-C cells six days after NIPBL depletion. Neurite-like structures were detected using the IncuCyte Live-Cell Neurite Analysis System. (**F**) Quantification of neurite outgrowth in BE(2)-C cells six days post-transfection, showing a significant increase in neurite extension following NIPBL depletion. Neurite length is normalized to cell-body cluster area. *p* < 0.05 (*), *p* < 0.01 (**). (**G**) Western blot analysis showing increased neuronal differentiation markers, MAP2 and TUBB3, upon NIPBL knockdown. GAPDH serves as a loading control.

We next investigated whether NIPBL regulates the MYCN-driven transcriptional network. From the BE(2)-C RNA-seq data, we observed that MYCN mRNA levels were significantly reduced upon NIPBL depletion (Figure 5A). Western blot analysis further confirmed a marked decrease in MYCN protein expression following NIPBL knockdown (Figure 5B). Given the reduction in MYCN expression, we examined whether NIPBL depletion also impacts MYCN-regulated transcriptional programs. Using previously published RNA-seq data from MYCN-depleted BE(2)-C cells [30], we found that 116 of the 868 genes downregulated by NIPBL knockdown overlapped with those downregulated upon MYCN depletion, and 303 of the 1297 genes upregulated by NIPBL knockdown overlapped with those upregulated following MYCN depletion (Figure 5C). These findings suggest that NIPBL is essential for regulating a substantial portion of the MYCN-driven transcriptome through maintaining MYCN mRNA and protein expression.

Using transcriptomic analyses of various MYCN-depleted NB cell lines, we identified gene sets representing “MYCN-activated canonical MYC targets,” including genes involved in ribosome biogenesis and RNA processing, as well as “MYCN-repressed neuronal genes,” encompassing pathways involved in neuronal morphogenesis and axon development [31]. These gene sets were used for GSEA of NIPBL-depleted RNA-seq data from BE(2)-C cells, which showed significant positive enrichment of MYCN-repressed gene sets associated with synaptic transmission, neuronal morphogenesis, and axon development (Figure 5D). These findings support a functional role for NIPBL in repressing neuronal differentiation programs through regulating MYCN expression.

We next tested whether NIPBL depletion induces neuronal differentiation in MYCN-amplified neuroblastoma cells. Live-cell imaging using IncuCyte ZOOM revealed the emergence of neurite-like extensions in NIPBL-depleted BE(2)-C cells (Figure 5E,F). Consistent with these findings, Western blot analysis confirmed increased expression of neuronal differentiation markers, including MAP2 and TUBB3, six days after NIPBL knockdown in BE(2)-C and IMR-32 cells (Figure 5G). A decrease in MYCN expression and a corresponding induction of neuronal differentiation were also observed in IMR-32 cells upon NIPBL depletion (Figure S2).

Together, these results suggest that NIPBL sustains the undifferentiated state of MYCN-amplified neuroblastoma cells by maintaining MYCN expression and supporting the MYCN-driven repression of neuronal differentiation programs.

## 4. Discussion

Our study demonstrates that NIPBL is aberrantly upregulated in neuroblastoma tumors compared to its normal counterparts, and its elevated expression is associated with unfavorable clinical outcomes. Functional studies revealed that NIPBL is essential for the proliferation of MYCN-amplified neuroblastoma cells, as its depletion significantly reduced cell viability across multiple cell lines. Transcriptomic profiling following NIPBL knockdown revealed widespread transcriptional changes, including the suppression of cell cycle–related pathways and activation of neuronal differentiation programs, suggesting that NIPBL maintains an undifferentiated, proliferative state. ChIP-seq analyses revealed that NIPBL co-occupies neuroblastoma-specific enhancers with MYCN, particularly at sites enriched for core regulatory circuitry (CRC) transcription factor motifs, suggesting a cooperative role in regulating lineage-specific transcription at neuroblastoma-specific enhancers. Mechanistically, NIPBL is required to sustain MYCN mRNA and protein expression, and its loss leads to transcriptional and phenotypic shifts toward neuronal differentiation. These findings establish NIPBL as a key upstream regulator of MYCN function and a critical factor in maintaining the oncogenic and undifferentiated phenotype of MYCN-amplified neuroblastoma.

Despite recent advances in treatment, outcomes for patients with high-risk neuroblastoma remain poor, underscoring the urgent need for targeted therapeutic strategies. MYCN, which is broadly expressed during neural crest cell migration and early differentiation, is typically downregulated during the terminal maturation of sympathetic neurons [32,33]. Its sustained expression disrupts this process, contributing to the undifferentiated and aggressive phenotype observed in high-risk neuroblastoma [33,34]. A defining molecular feature of high-risk disease is MYCN amplification, which drives widespread transcriptional dysregulation. Our previous work showed that MYCN activates genes related to self-renewal and stemness by binding to promoters containing canonical MYC motifs. In contrast, MYCN amplification promotes enhancer invasion at non-canonical E-box binding motifs, leading to repression of genes involved in neuronal differentiation [31]. Targeting MYCN-driven transcriptional networks, therefore, holds promise for shifting tumor cells toward a less aggressive, more differentiated state.

A hallmark of neuroblastoma is the failure of sympathoadrenal progenitor cells to undergo proper differentiation, leading to persistent self-renewal. Poor prognosis is strongly associated with undifferentiated or stroma-poor tumors, whereas more differentiated tumor phenotypes are linked to favorable clinical outcomes and spontaneous regression [32]. Analysis of patient tumor datasets revealed that NIPBL expression is significantly elevated in neuroblastoma tumors compared to normal adrenal tissue and neural crest-derived precursors. Interestingly, NIPBL expression was also higher in neural crest-derived precursors than in differentiated adrenal tissue, suggesting that elevated NIPBL expression is associated with an undifferentiated cellular phenotype. Although NIPBL levels were not associated with conventional risk classifications or MYCN amplification status, high NIPBL expression was strongly correlated with poor overall and event-free survival.

NIPBL depletion resulted in impaired neuroblastoma cell proliferation, accompanied by global transcriptional reprogramming. Downregulated genes were enriched in cell cycle and mitotic pathways, while upregulated genes were associated with synaptic transmission, axonogenesis, and neuronal morphogenesis—features consistent with neuronal differentiation. These transcriptional changes were paralleled by the induction of neuronal markers and morphological signs of neuronal differentiation, indicating that NIPBL is essential for maintaining the undifferentiated, stem-like state of MYCN-amplified neuroblastoma cells.

Our findings reveal a critical role for NIPBL in shaping the MYCN-driven transcriptional landscape of high-risk neuroblastoma. While MYCN broadly binds to both promoters and distal regulatory elements, we find that a subset of these regions, co-occupied by NIPBL, is functionally linked to lineage-specific developmental programs, particularly those governing the autonomic nervous system. These NIPBL-bound MYCN peaks are highly enriched for motifs of core regulatory circuitry (CRC) transcription factors such as GATA3, PHOX2A, HAND1, and HAND2, which contribute to the maintenance of the neuroblastoma oncogenic cell identity. CRC transcription factors bind with MYCN at cell-type-specific super-enhancers, forming an interconnected, self-reinforcing transcriptional network that governs the neuroblastoma-specific transcriptome [35]. In contrast, MYCN peaks lacking NIPBL are associated with general biosynthetic and stress-responsive processes, including mRNA metabolism, protein targeting, and viral gene expression, suggesting a broader and more context-independent function of MYCN at these loci. Importantly, NIPBL also colocalizes with transcription factors involved in neuronal differentiation at MYCN-independent enhancer regions, suggesting a dual role for NIPBL in both sustaining an undifferentiated, proliferative neuroblastoma state and modulating lineage-specific gene expression programs. These findings indicate the selective enhancer recruitment of NIPBL may be a mechanism by which MYCN activity is spatially and functionally diversified. They also raise the possibility that NIPBL is a potential regulator of neuroblastoma cell plasticity and differentiation.

Importantly, NIPBL depletion led to the activation of MYCN-repressed neuronal differentiation genes, which significantly overlapped with those upregulated upon MYCN knockdown. This indicates that NIPBL not only regulates MYCN expression but also facilitates its transcriptional output. Our results support a model in which NIPBL promotes MYCN binding at enhancers and sustains its repressive influence on neuronal differentiation programs, thereby reinforcing the MYCN-driven oncogenic transcriptional network that maintains the neuroblastoma phenotype.

In summary, our study establishes NIPBL as a central regulator of MYCN-driven transcription in neuroblastoma. By supporting MYCN expression and enhancer occupancy, NIPBL facilitates the repression of neuronal differentiation programs driven by MYCN, thereby inhibiting the differentiation of neuroblastoma. These findings position NIPBL as a potential therapeutic vulnerability, especially given that neuroblastoma differentiation is associated with improved clinical outcomes. Since differentiation-promoting agents can sensitize tumors to chemotherapy, future strategies may benefit from combining NIPBL inhibition with pro-differentiation therapies. In addition, NIPBL has been shown to interact with BRD4, a member of the bromodomain and extraterminal domain (BET) protein family. Loss of BRD4 disrupts genome folding and impairs neural crest differentiation, in part by reducing NIPBL chromatin occupancy [36]. These findings suggest that NIPBL function could be attenuated indirectly through modulation of BRD4 activity, thereby suppressing the MYCN-driven oncogenic transcriptome. Supporting this possibility, previous studies have shown that BRD4 inhibition reduces MYCN expression in neuroblastoma cells [37]. Together, these observations raise the potential for therapeutically targeting the NIPBL–MYCN axis through BRD4 inhibition.

Nonetheless, important questions remain. It is still unclear whether the regulatory relationship between NIPBL and MYCN involves direct physical interaction or is mediated indirectly through broader effects on cohesin loading and chromatin topology. Further investigation is needed to dissect these mechanisms, including 3D chromatin mapping and comparative analyses in MYCN-nonamplified models. Given the growing interest in targeting transcriptional dependencies in MYCN-amplified cancers, therapeutic inhibition of NIPBL will offer a promising strategy to disrupt MYCN-driven oncogenic networks and restore differentiation potential in high-risk neuroblastoma.

## 5. Conclusions

Our study reveals that the cohesin loading factor NIPBL plays a pivotal role in maintaining the MYCN-driven oncogenic transcriptional program in high-risk neuroblastoma. Elevated NIPBL expression correlates with poor clinical outcomes and an undifferentiated tumor phenotype. Mechanistically, NIPBL co-occupies neuroblastoma-specific enhancers with MYCN, particularly at regulatory regions enriched for core regulatory circuitry (CRC) transcription factor binding motifs. This suggests that NIPBL co-occupies MYCN-bound enhancers, potentially supporting MYCN-driven transcriptional programs. However, further studies are required to determine whether NIPBL directly stabilizes MYCN binding to these elements. We further demonstrate that NIPBL is essential for sustaining MYCN mRNA and protein expression, thereby supporting the MYCN-driven oncogenic transcriptome. Loss of NIPBL induces widespread transcriptional reprogramming characterized by reduced proliferation and increased expression of neuronal differentiation markers, indicating a shift toward a more differentiated state. These findings position NIPBL as a critical upstream regulator of MYCN function, identifying it as a potential therapeutic vulnerability. Targeting NIPBL may offer a novel strategy to disrupt MYCN-driven transcription and promote tumor cell differentiation in MYCN-amplified neuroblastoma, ultimately improving therapeutic outcomes in this aggressive pediatric cancer.

## Figures and Tables

**Table 1:** Antibodies.

Target	Company	Cat	Host	Application	Dilution	Lot
NIPBL	Bethyl	A301-779A	Rabbit	WB	1000 or 500	4
MYCN	Santa Cruz Biotechnologies	sc-53993	Mouse	WB	1000	B2316
GAPDH	Santa Cruz Biotechnologies	Sc-47727	Rabbit	WB	1000	B2719
anti-rabbit IgG-HRP	CellSignaling Technologies	7074S	Rabbit	WB	500	29
goat anti-rabbit IgG-HRP	Santa Cruz Biotechnologies	Sc-2004	Goat	WB	500	B2213
mouse anti-rabbit IgG-HRP	Santa Cruz Biotechnologies	Sc-2357	Mouse	WB	1000	D0423
anti-mouse IgG-HRP	Santa Cruz Biotechnologies	Sc-525409	Mouse	WB	500	D2523

## Data Availability

The data generated in this study are available within the manuscript and its Supplementary Materials. RNA-seq of MYCN silencing for 72 hours in BE(2)-C can be found under GEO accession number GSE120859.

## Supplementary Materials

The following supporting information can be downloaded at: https://www.mdpi.com/article/10.3390/cancers17162615/s1, Figure S1: NIPBL is required for the proliferation of MYCN-amplified neuroblastoma cells, KCRN and IMR-32; Figure S2: NIPBL regulates MYCN expression and represses neuronal differentiation programs in IMR-32.
