# Supplementary figures and images for: Cohesin Loading Factor NIPBL Is Essential for MYCN Expression and MYCN-Driven Oncogenic Transcription in Neuroblastoma
Source: Cancers (Basel). 2025 Aug 9;17(16):2615. doi: 10.3390/cancers17162615 (PMC12384541; doi:10.3390/cancers17162615)

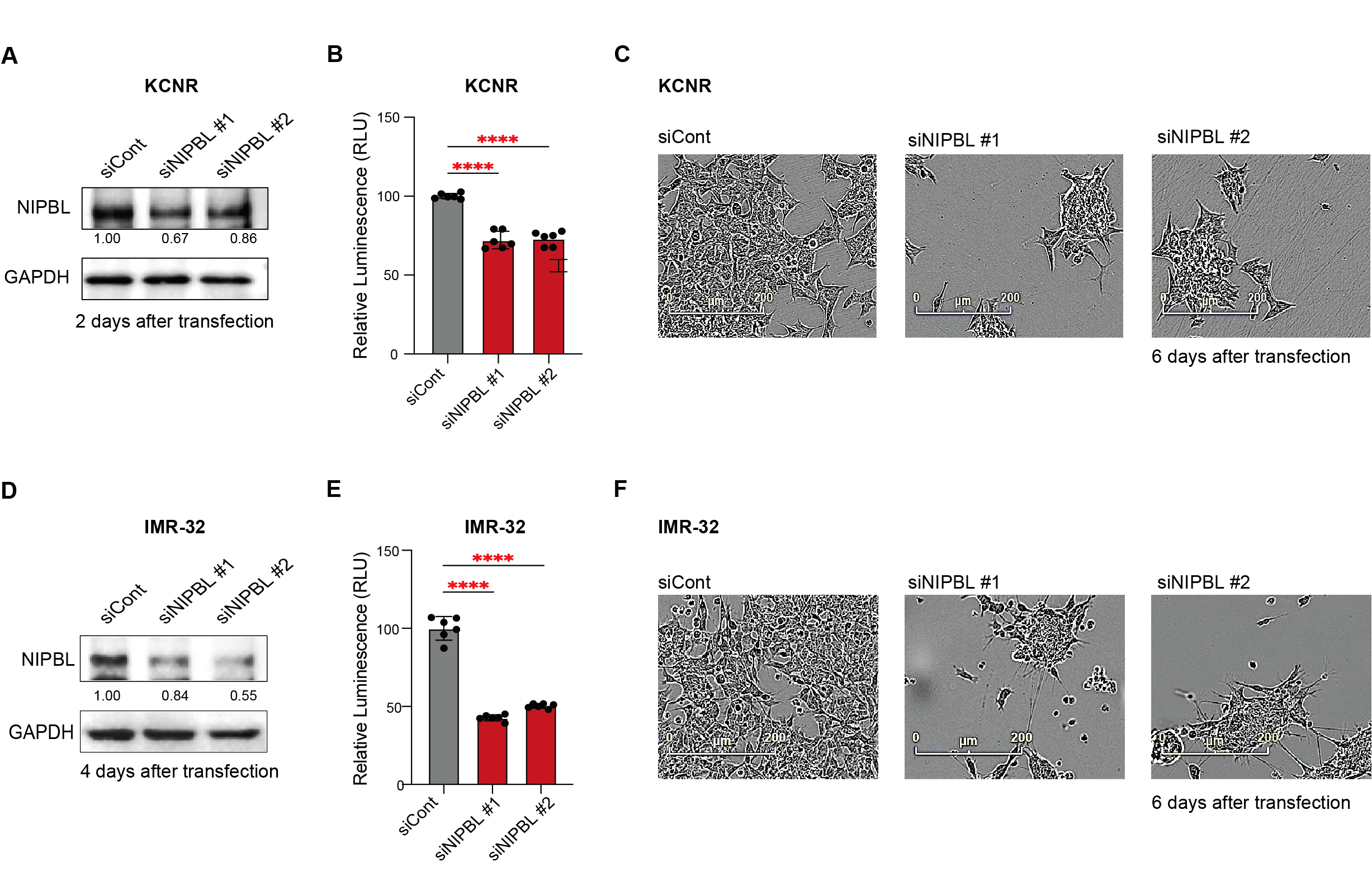

Supplement: Supplementary file 1 [file cancers-17-02615-s001.zip › Supplementary Figure_S1.png]

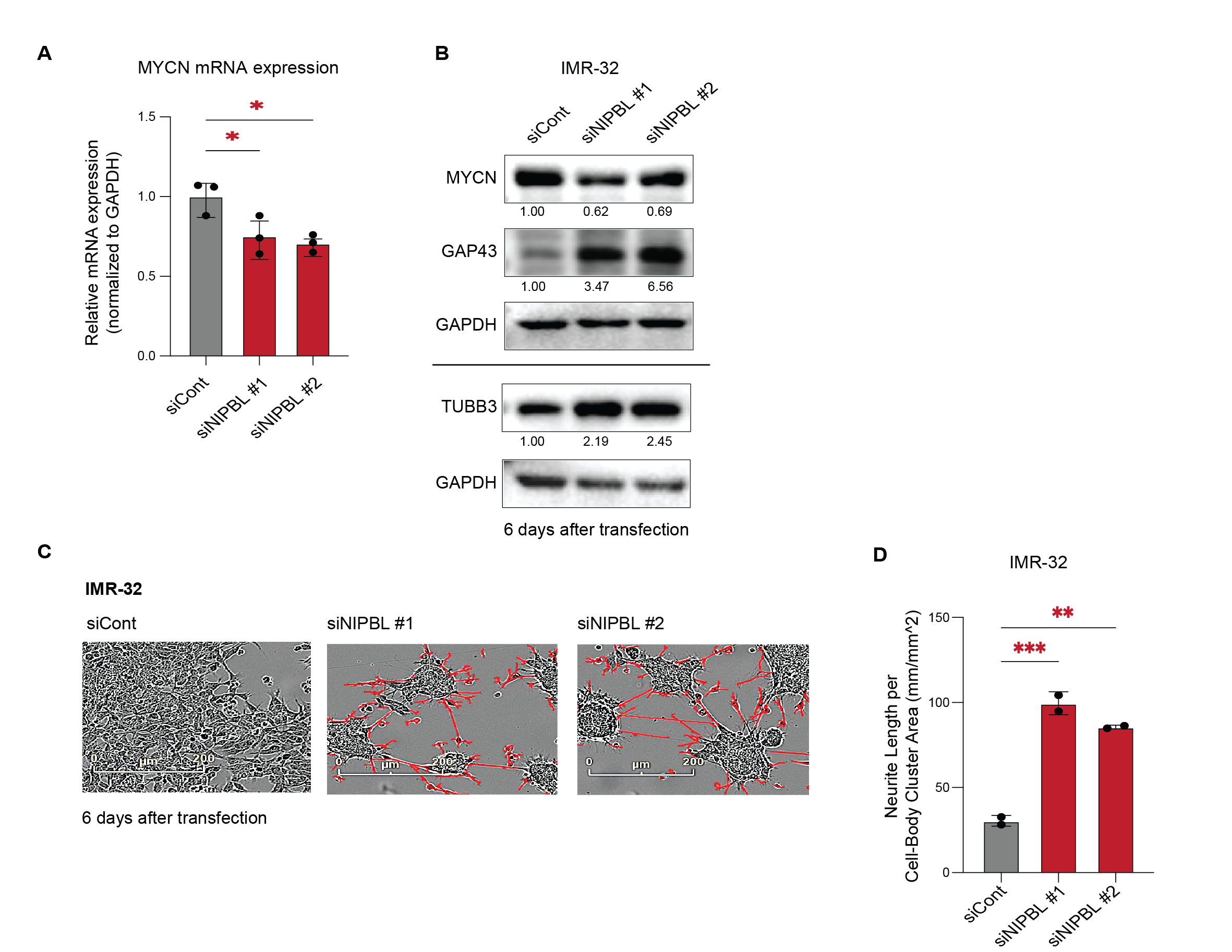

Supplement: Supplementary file 1 [file cancers-17-02615-s001.zip › Supplementary Figure_S2.png]
